# Do the instruments used to assess fibromyalgia symptoms according to American College of Rheumatology criteria generate similar scores in other chronic musculoskeletal pain?

**DOI:** 10.1186/s12891-023-06572-x

**Published:** 2023-06-07

**Authors:** André Pontes-Silva, Ana Paula de Sousa, Almir Vieira Dibai-Filho, Marcelo Cardoso de Souza, Josimari Melo DeSantana, Mariana Arias Avila

**Affiliations:** 1 Study Group on Chronic Pain (NEDoC), Laboratory of Research on Electrophysical Agents (LAREF), Physical Therapy Department, Universidade Federal de São Carlos, Rodovia Washington Luís, Km 235, São Carlos, São Paulo, 13565-905 Brazil; 2 Postgraduate Program in Physical Education, Department of Physical Education, Universidade Federal do Maranhão, São Luís, MA Brazil; 3 Postgraduate Program in Rehabilitation Sciences. Physical Therapy Department, Universidade Federal do Rio Grande do Norte, Natal, RN Brazil; 4 Laboratory of Research on Neuroscience (LAPENE), Physical Therapy Department, Graduate Program in Health Science, Graduate Program in Physiological Science, Universidade Federal de Sergipe, Aracaju, Sergipe Brazil

**Keywords:** Chronic Pain, Diagnostic Services, Rheumatology, Primary Health Care

## Abstract

**Background:**

As with fibromyalgia, several musculoskeletal disorders are characterized by chronic pain, raising a clinical question – do the instruments used to assess fibromyalgia symptoms according to ACR criteria (ACR criteria) generate similar scores in other chronic musculoskeletal pain?

**Objective:**

To compare the symptoms among fibromyalgia and other chronic musculoskeletal pain. Additionally, we also compared the most researched outcomes in fibromyalgia (i.e., present pain at rest and after movement; fatigue; pain severity and impact; function, global impact, and fibromyalgia symptom).

**Methods:**

A cross-sectional study. Participants over 18 years old were included if they presented report of chronic musculoskeletal pain (≥ 3 months) and after that, they were divided into two groups (fibromyalgia and chronic pain). They answered the Fibromyalgia Impact Questionnaire-Revised (FIQ-R), Brief Pain Inventory (BPI), Numerical Pain Rating Scale (NPRS) for pain and fatigue, WPI, and SSS.

**Results:**

A total of 166 participants were included in this study into two independent groups (chronic pain, n = 83; fibromyalgia, n = 83). We observed significant differences (p < 0.05) and large effect sizes (Cohen’s d, ≥ 0.7) in clinical outcomes comparisons between groups (i.e., widespread pain; symptom severity; present pain at rest and after movement; fatigue; pain severity and impact; function, global impact, and fibromyalgia symptoms).

**Conclusion:**

Fibromyalgia patients (2016 ACR criteria) compared to other chronic musculoskeletal pain patients have higher levels of pain (at rest or after movement) and fatigue, greater impairment in both functionality and global impact, and worse symptoms. Therefore, the WPI and SSS instruments should be used exclusively to assess fibromyalgia symptoms.

**Supplementary Information:**

The online version contains supplementary material available at 10.1186/s12891-023-06572-x.

## Introduction

Fibromyalgia (or fibromyalgia syndrome) is a chronic condition characterized by widespread pain and complex symptomatology [1], such as fatigue [2], sleep disturbances [3], mood disorders [4], and symptoms not explained by structural changes [5]. Its prevalence varies from 2 to 6% in the world population and is more identified in women aged between 20 and 55 years [2]. The literature recommends that the diagnosis of fibromyalgia should be based on the guidelines of the American College of Rheumatology (ACR) [6]. However, taking into account that it is a disease characterized (in part) by chronic musculoskeletal pain, nothing prevents us from using the same ACR criteria to evaluate other musculoskeletal diseases, as the fibromyalgia assessment/diagnosis (2016 ACR criteria) uses generic instruments such as the Widespread Pain Index (WPI) and the Symptom Severity Scale (SSS) [6].

However, although WPI and SSS instruments perform generic assessments of chronic musculoskeletal pain (on axial region and/or upper and lower limbs) [6], it is necessary to verify whether there is a difference among the clinical outcomes’ specificities (e.g., function, global impact, symptoms, pain severity/impact, and fatigue) when comparing patients with fibromyalgia to patients with other chronic musculoskeletal pain. Therefore, the hypothesis of this study was that patients with fibromyalgia have a worse clinical outcome compared to other chronic musculoskeletal pain.

Thus, the objective of this study was to compare (via WPI and the SSS) the symptoms among fibromyalgia and other chronic musculoskeletal pain. Additionally, we also compared the most researched outcomes in fibromyalgia (i.e., present pain at rest and after movement [7, 8]; fatigue [7, 8]; pain severity and impact [9]; function, global impact, and fibromyalgia symptom [10]).

## Methods

### Study design and ethical considerations

A cross-sectional study performed according to the STROBE Guidelines [11]. The protocol has been approved by the ethics committee of Universidade Federal de São Carlos, in Brazil (report number 4.193.940). This study was disclosed in social media from November 2020 to August 2021.

We disclosed in social media (Instagram® and Facebook®) and through messaging applications (WhatsApp®). All people who manifested interest in taking part in the study were contacted and checked for eligibility criteria. All those who were included in the study received an online form (via GoogleForms®) and agreed to take part in the study by clicking on the “I agree to take part in the present study” after reading the informed online consent form. All participants received an online booklet with information regarding fibromyalgia / chronic pain after the end of their participation (Supplementary material 1).

### Participants and study size

Considering the primary outcome of our study (comparisons between groups), we performed the sample calculation *a priori* using the t-test two tails (independent groups) through G*Power (version 3.1.9.7). We used the effect size of 0.44 [12, 13], alpha of 0.05, power of 0.80, and critical f of 1.97. As such, the total sample size was estimated at 166 participants divided into 2 independent groups [14] (fibromyalgia, n = 83; chronic pain, n = 83).

Participants over 18 years old that could read and write in Brazilian Portuguese were included if they presented report of chronic musculoskeletal pain (≥ 3 months) and after that, they were divided into two groups (fibromyalgia and chronic pain). For the fibromyalgia group, people should have the fibromyalgia diagnosis (participants were considered as with fibromyalgia if they fulfilled the ACR 2016 fibromyalgia diagnostic criteria [6], including the WPI ≥ 7 and the SSS ≥ 5 or WPI = 4–6 and SSS ≥ 9). For the chronic pain group, participants should have a history of chronic pain (≥ 3 months) but no fibromyalgia (i.e., arthritis, osteoarthritis, low back pain, neck pain, and so on). Namely, participants with chronic pain whose symptoms do not meet the ACR 2016 fibromyalgia diagnostic criteria. Complete database is available at the link < https://docs.google.com/spreadsheets/d/1Yxno-f1JH0bUEbD44ZfxBx8ILqcjg58Z/edit?usp=sharing&ouid=104821689851272179944&rtpof=true&sd=true>.

Participants were excluded from the analysis if they had a history of tumors, traumas, acute infections, self-report of severe psychiatric illnesses (including severe depression, bipolarity, and schizophrenia), presence of severe comorbidities in the heart, liver, and/or kidney, presence of neoplasia, systemic autoimmune or inflammatory concomitant diseases, hypothyroidism, pregnancy and/or breastfeeding, and presence of therapeutic intervention in the last six months.

### Assessment tools

Participants answered Fibromyalgia Impact Questionnaire-Revised (FIQ-R) [10], Brief Pain Inventory (BPI) [9], and Numerical Pain Rating Scale (NPRS) [7, 8] for pain and fatigue. All participants rated their pain (in two different situations: at rest, and after body movement) and fatigue from 0 (if they did not present pain/fatigue) to 10 (if they presented the worst imaginable pain or fatigue).

FIQ-R assesses the impact of fibromyalgia on life in relation to functional capacity, professional status, psychological disorders, and physical symptoms [15]. Its Brazilian version has excellent test-retest reliability (ICC = 0.75) and comprises 21 items that investigate three domains: function (9 items, 30 points), global impact (2 items, 20 points), and symptoms (10 items, 50 points) [10, 15]. Scores range from 0 to 100, with the latter being meaningful of a worst condition. The minimal important clinical difference for the FIQ-R is 27 points [16].

We also use BPI, a self-report instrument validated for the Brazilian population [17]. BPI assesses pain severity and impact on a person’s life with 15 items that assess presence, severity, location, functional impact, used therapeutic strategies, and treatment efficacy on an 11-point scale ranging from zero (no pain/no interference) to 10 (as bad as it can be). High scores indicate worse pain severity and impact. Its Brazilian version presented a two-dimensional structure (pain severity and interference) and excellent internal consistency (α = 0.87–0.91) [9].

We assess pain intensity using the NPRS, a self-report instrument validated for the Brazilian population [7]. NPRS is a single-item instrument that was used for pain and fatigue intensity. We evaluated pain in two different situations: at rest – “Currently and at the moment when you are sitting/lying on the couch watching your favorite TV show, do you feel pain?”; after body movement – “Currently and when you walked from the supermarket parking lot to the grocery store or crossed the street to work, do you feel pain?” [18]. For fatigue, we asked “During the answer to this questionnaire, which number best corresponds to your state of fatigue/body tiredness?“. In all questions about pain/fatigue, zero means no pain/fatigue and 10 was the worst pain/fatigue imaginable. In chronic pain conditions, NPRS had a moderate to high test-retest reliability (0.67 to 0.96) [19].

### Statistical analysis

The distribution of variables was verified using Kolmogorov-Smirnov test. We set the significance level at 5% for all statistical tests, which in turn were processed using the Statistical Package for the Social Sciences software, version 17.0 (Chicago, IL, USA). The comparisons between variables were performed via independent T-test and presented as: mean, standard deviation (SD), mean difference (MD) with confidence interval (95% CI), and effect size [20], calculated using Cohen’s d according to classification values: 0.2 = small, 0.5 = moderate, and ≥ 0.7 large [21].

## Results

Three hundred and ninety-six women volunteered. After the screening, according to the eligibility criteria mentioned in the methods, a total of 166 participants were included in this study into two independent groups (Chronic Pain [CP], n = 83; Fibromyalgia [FM], n = 83). We observed significant differences (p < 0.05) and large effect sizes (Cohen’s d, ≥ 0.7) in clinical outcomes comparisons shown in Tables 1 and 2 (i.e., widespread pain [WPI]; symptom severity [SSS]; present pain at rest, after movement, and fatigue [NPRS]; pain severity and impact [BPI]; function, global impact, and fibromyalgia symptoms [FIQ-R]). Prevalence of WPI is similar in both groups. However, the number of regions affected by pain is significantly different between them (Table 1).

**Table 1:** Participants’ characteristics – values presented in mean (SD).

Variables	Chronic pain group (n = 83)	Fibromyalgia group (n = 83)	p
Age (years)	43.1 (13.0)	38.7 (10.1)	0.011*
Body mass (kg)	71.7 (13.5)	73.5 (15.5)	0.085
Stature (m)	1.62 (0.0)	1.64 (0.0)	0.766
Body mass index (Kg/m^2^)	27.3 (5.2)	27.2 (5.3)	0.795
Widespread Pain Index (score)	4.8 (2.7)	13.3 (3.6)	< 0.001*
Symptom Severity Scale (score)	5.9 (2.9)	9.2 (1.9)	< 0.001*
Numerical Pain Rating Scale (score)			
Present pain at rest	5.0 (2.5)	6.6 (1.8)	0.007*
Pain after movement	5.4 (2.7)	7.5 (2.2)	0.017*
Present fatigue	5.2 (3.3)	7.9 (1.8)	< 0.001*
Brief Pain Inventory (score)			
Pain severity	5.2 (2.2)	6.8 (1.6)	< 0.001*
Pain impact	5.5 (2.7)	7.4 (2.0)	0.001*
FIQ-R (score)			
Function (0–30)	11.9 (8.6)	21.5 (5.9)	< 0.001*
Global impact (0–20)	9.5 (6.3)	16.3 (3.8)	< 0.001*
Symptoms (0–50)	25.8 (12.3)	37.6 (6.9)	< 0.001*
Total score (0-100)	47.4 (25.5)	75.5 (14.6)	< 0.001*

BPI: Brief Pain Inventory; FIQ-R: Fibromyalgia Impact Questionnaire-Revised. * Significant difference (independent t-test, p < 0.05)

**Table 2:** Comparison of Pain and FIQ-R between groups

Variables	Group (n = 166/2)	Mean (SD)	Mean Difference	CI 95%	d
NPRS^a^	Chronic Pain	5.0 (2.5)	-1.62	-2.30, -0.95	0.7^#^
	Fibromyalgia	6.6 (1.8)			
NPRS^b^	Chronic Pain	5.4 (2.7)	-2.12	-2.87, -1.36	0.8^#^
	Fibromyalgia	7.5 (2.2)			
NPRS^c^	Chronic Pain	5.2 (3.3)	-2.66	-3.49, -1.82	1.0^#^
	Fibromyalgia	7.9 (1.8)			
Brief Pain Inventory					
Pain severity	Chronic Pain	5.2 (2.2)	-1.50*	-2.18, -0.99	0.8^#^
	Fibromyalgia	6.8 (1.6)			
Pain impact	Chronic Pain	5.5 (2.7)	-1.95*	-2.68, -1.22	0.8^#^
	Fibromyalgia	7.4 (2.0)			
FIQ-R					
Function	Chronic Pain	11.9 (8.6)	-9.57*	-11.86, 7.29	1.3^#^
	Fibromyalgia	21.5 (5.9)			
Global impact	Chronic Pain	9.5 (6.3)	-6.77*	-8.38, -5.15	1.3^#^
	Fibromyalgia	16.3 (3.8)			
Symptoms	Chronic Pain	25.8 (12.3)	-11.80*	-14.87, -8.73	1.1^#^
	Fibromyalgia	37.6 (6.9)			
Total score	Chronic Pain	47.4 (25.5)	-28.15*	-34.53, 21.77	1.3^#^
	Fibromyalgia	75.5 (14.6)			

CI: Confidence Interval; d: effect size (Cohen’s d); FIQ-R: Fibromyalgia Impact Questionnaire-Revised; NPRS: Numerical Pain Rating Scale (^a^ present pain at rest, ^b^ pain after movement, ^c^ present fatigue); SD: Standard Deviation. * Significant difference (independent t-test, p < 0.05). ^#^ Significant effect size (Cohen’s d, large effect, ≥ 0.8)

Our study shows large effect sizes, indicating the clinical relevance of the comparisons of the present study. Clinical relevance (also known as clinical significance) indicates that the results of a study are meaningful or not for several stakeholders [22]. The clinical relevance facilitates the understanding and interpretation of results for professionals. Currently, the assessment of this approach has become a popular method to assist the transfer of knowledge into clinical practice.

## Discussion

### Main results synthesis

The objective of this study was to compare (via WPI and the SSS) the symptoms among fibromyalgia and other chronic musculoskeletal pain. Additionally, we also compared the most researched outcomes in fibromyalgia (i.e., present pain at rest and after movement [7, 8]; fatigue [7, 8]; pain severity and impact [9]; function, global impact, and fibromyalgia symptom [10]). Our results showed that patients with fibromyalgia have higher levels of widespread pain and symptom severity than patients with other chronic musculoskeletal pain. Namely, the instruments used to assess fibromyalgia symptoms according to ACR criteria (WPI + SSS) generate significantly different scores in other chronic musculoskeletal pain (the same happens with the other outcomes analyzed). Therefore, reinforcements that these instruments (WPI + SSS) should be used only in patients with fibromyalgia.

### Fibromyalgia diagnosis: challenges and perspectives

Since the last century, studies on fibromyalgia have evaluated patients using biomedical models [23, 24]. This evaluation model was strengthened 1990’s year when the ACR established classification criteria for fibromyalgia [25]. Then, other updates appeared (2010 and 2011) whose combined review resulted in the 2016 criteria [26]: (A) Widespread pain, defined as pain in at least 4 of 5 body’s regions; (B) Symptoms have been present at a similar level for at least three months; (C) Widespread pain index ≥ 7 and symptom severity scale score ≥ 5 (or Widespread pain index of 4–6 and symptom severity scale score ≥ 9); (D) A diagnosis of fibromyalgia is valid irrespective of other diagnoses [6].

Although this initiative is relevant to health sciences, it is possible to note that patients continue to be assessed via the biomedical model [27]. Perhaps, that happening because The prevalence of fibromyalgia appears to differ according to the diagnostic criteria used [28]. The 1990 criteria have been considered stricter than the 2010 criteria, such that only more severely affected patients are identified [29]. Studies recruiting fibromyalgia patients according to the 1990 ACR criteria reported higher mean WPI and SSS scores than studies in which patients were recruited using the 2010 ACR criteria [29, 30]. However, most recent studies as well as international recommendations guide the use of the 2016 ACR criteria [6].

### Ours and the literature’s results

Chronic musculoskeletal pain is one of the main reasons for referrals to health professionals [31]. It can be caused by a wide variety of inflammatory [32] and noninflammatory conditions [33], including arthritis [34], hypermobility [35], low back pain [32], neck pain [36], growing pains [35], and complex regional pain syndrome [37]. Some patients with fibromyalgia have these symptoms associated with the disease, thus, hindering the diagnostic accuracy of fibromyalgia [28]. As such, some authors have used the WPI in the evaluation of other disorders, e.g., temporomandibular disorders [28], psoriatic arthritis [38], musculoskeletal surgery [39], and headache [39].

Our results indicate that the WPI, as well as the SSS, should be used exclusively on fibromyalgia evaluation. In addition, the scores of the instruments most used in studies on fibromyalgia (FIQ-R, BPI, and NPRS), observed in our study, reinforced that the severity of symptoms is greater in fibromyalgia patients. However, although our results support the use of SSS in patients with fibromyalgia, we highlighted that Elkana et al. [40] found that the SSS has an insignificant relationship between the subjective appraisal of cognitive impairment and the objective cognitive scores on computerized subtests. Therefore, we suggest novel studies in this area.

### Strengths and clinical applicability

Although patients with fibromyalgia have chronic musculoskeletal pain in different parts of the body (spine, knee, and so on [1]), it does not mean that patients who have other chronic musculoskeletal pain, but no fibromyalgia, may be evaluated using the same instruments proposed by the ACR to assess fibromyalgia symptoms (WPI and SSS) [6].

As clinical applicability to evidence-based practice, we suggest to the health professionals, first of all, screen fibromyalgia using specific instruments (e.g., the fibromyalgia rapid screening tool [41]). In the same way, other chronic musculoskeletal pain must be evaluated by specific instruments (respecting the cross-cultural adaptation [42]), because there are questionnaires, scales, and specific tests for low back pain [43], knee pain [44], neck pain [45], temporomandibular disorders [46], and other musculoskeletal diseases. Therefore, the WPI and SSS apply to the individuals which ACR has suggested: fibromyalgia patients [1, 6, 47].

### Limitations and prospects for novel studies

Although this was the first study to compare fibromyalgia symptoms, according to ACR criteria (WPI + SSS), to scores in other chronic musculoskeletal pain, our study has limitations that must be addressed. Although participants reported pain and global symptoms lasting > 3 months, we do not know the pain duration total values in the groups, the prevalence of headache, cramps in the lower abdomen, and depression during the previous six months. We also do not know if the investigated instruments (WPI + SSS) are sufficient to assess fibromyalgia symptoms or whether they should be associated with computerized tests. Besides, medication use was not controlled in that study, and we did not use a polysymptomatic distress scale— we suggest novel studies to investigate these limitations.

## Conclusion

Fibromyalgia patients (2016 ACR criteria) compared to other chronic musculoskeletal pain patients have higher levels of pain (at rest or after movement) and fatigue, greater impairment in functionality and global impact, and worse symptoms. Therefore, WPI and SSS instruments should be used exclusively to assess fibromyalgia symptoms.

## Electronic supplementary material

Below is the link to the electronic supplementary material.

Supplementary Material 1

## Data Availability

The data and materials in this paper are available from the corresponding author on request.

## List of abbreviations

ACR: American College of Rheumatology
BPI: Brief Pain Inventory
FIQ-R: Fibromyalgia Impact Questionnaire-Revised
MD: Mean Difference
NPRS: Numerical Pain Rating Scale
SD: Standard-Deviation
SSS: Symptom Severity Scale
WPI: Widespread Pain Index

## References

[1] Macfarlane GJ, Kronisch C, Dean LE, Atzeni F, Häuser W, Flub E (2017). EULAR revised recommendations for the management of fibromyalgia. Ann Rheum Dis.

[2] Sarzi-Puttini P, Giorgi V, Marotto D, Atzeni F (2020). Fibromyalgia: an update on clinical characteristics, aetiopathogenesis and treatment. Nat Rev Rheumatol.

[3] Ughreja RA, Venkatesan P, Balebail Gopalakrishna D, Singh YP (2021). Effectiveness of myofascial release on pain, sleep, and quality of life in patients with fibromyalgia syndrome: a systematic review. Complement Ther Clin Pract.

[4] Valencia C, Fatima H, Nwankwo I, Anam M, Maharjan S, Amjad Z (2022). A correlation between the pathogenic processes of Fibromyalgia and irritable bowel syndrome in the Middle-Aged Population: a systematic review. Cureus.

[5] Kudlow PA, Rosenblat JD, Weissman CR, Cha DS, Kakar R, McIntyre RS (2015). Prevalence of fibromyalgia and co-morbid bipolar disorder: a systematic review and meta-analysis. J Affect Disord.

[6] Wolfe F, Clauw DJ, Fitzcharles MA, Goldenberg DL, Häuser W, Katz RL (2016). 2016 revisions to the 2010/2011 fibromyalgia diagnostic criteria. Semin Arthritis Rheum.

[7] Ferreira-Valente MA, Pais-Ribeiro JL, Jensen MP (2011). Validity of four pain intensity rating scales. Pain.

[8] Farrar JT, Young JP, LaMoreaux L, Werth JL, Poole RM (2001). Clinical importance of changes in chronic pain intensity measured on an 11-point numerical pain rating scale. Pain.

[9] Ferreira KA, Teixeira MJ, Mendonza TR, Cleeland CS (2011). Validation of brief pain inventory to brazilian patients with pain. Support Care Cancer.

[10] Lupi JB, Carvalho de Abreu DC, Ferreira MC, de Oliveira RDR, Chaves TC (2017). Brazilian portuguese version of the revised Fibromyalgia Impact Questionnaire (FIQR-Br): cross-cultural validation, reliability, and construct and structural validation. Disabil Rehabil.

[11] Malta M, Cardoso LO, Bastos FI, Magnanini MMF, da Silva CMFP (2010). STROBE initiative: guidelines on reporting observational studies. Rev Saude Publica.

[12] Kundakci B, Kaur J, Goh SL, Hall M, Doherty M, Zhang W (2022). Efficacy of nonpharmacological interventions for individual features of fibromyalgia: a systematic review and meta-analysis of randomised controlled trials. Pain.

[13] Serrat M, Sanabria-Mazo JP, Almirall M, Musté M, Feliu-Soler A, Méndez-Ulrich JL et al. Effectiveness of a Multicomponent Treatment based on Pain Neuroscience Education, Therapeutic Exercise, cognitive behavioral therapy, and mindfulness in patients with Fibromyalgia (FIBROWALK Study): a Randomized Controlled Trial. Phys Ther. 2021;101. 10.1093/ptj/pzab200 34499174

[14] Kang H (2021). Sample size determination and power analysis using the G*Power software. J Educ Eval Health Prof.

[15] Bennett RM, Friend R, Jones KD, Ward R, Han BK, Ross RL (2009). The revised Fibromyalgia Impact Questionnaire (FIQR): validation and psychometric properties. Arthritis Res Ther.

[16] Surendran S, Mithun CB. FRI0647 estimation of minimum clinically important difference in fibromyalgia for fiqr using bpi as the anchor measure. FRIDAY, 15 JUNE 2018. BMJ Publishing Group Ltd and European League Against Rheumatism; 2018. 8451–845.

[17] Ferreira KA, Teixeira MJ, Mendonza TR, Cleeland CS (2011). Validation of brief pain inventory to brazilian patients with pain. Support care cancer Off J Multinatl Assoc Support Care Cancer.

[18] Avila MA, Camargo PR, Ribeiro IL, Zamunér AR, Salvini TF (2014). Three-dimensional scapular motion during arm elevation is altered in women with fibromyalgia. Clin Biomech.

[19] Kahl C, Cleland JA (2005). Visual analogue scale, numeric pain rating scale and the McGill pain questionnaire: an overview of psychometric properties. Phys Ther Rev.

[20] Pontes-Silva A (2022). Statistical significance does not show clinical relevance: we need to go beyond the P-value. J Clin Exp Hepatol.

[21] Cohen J. Statistical power analysis for the behavioral sciences. Academic Press; 1977.

[22] Armijo-Olivo S (2018). The importance of determining the clinical significance of research results in physical therapy clinical research. Brazilian J Phys Ther.

[23] Yunus M, Masi AT, Calabro JJ, Miller KA, Feigenbaum SL (1981). Primary fibromyalgia (fibrositis): clinical study of 50 patients with matched normal controls. Semin Arthritis Rheum.

[24] Smythe HA, Moldofsky H (1977). Two contributions to understanding of the “fibrositis” syndrome. Bull Rheum Dis.

[25] Wolfe F, Smythe HA, Yunus MB, Bennett RM, Bombardier C, Goldenberg DL (1990). The American College of Rheumatology 1990 Criteria for the classification of Fibromyalgia. Report of the Multicenter Criteria Committee. Arthritis Rheum.

[26] Wolfe F, Häuser W (2011). Fibromyalgia diagnosis and diagnostic criteria. Ann Med.

[27] Pontes-Silva A, Fibromyalgia (2023). Are we using the biopsychosocial model?. Autoimmun rev.

[28] Galvez-Sánchez CM. Reyes Del Paso GA. Diagnostic criteria for Fibromyalgia: critical review and future perspectives. J Clin Med. 2020;9. 10.3390/jcm9041219 PMC7230253 32340369

[29] Wolfe F, Clauw DJ, Fitzcharles M-A, Goldenberg DL, Katz RS, Mease P (2010). The American College of Rheumatology preliminary diagnostic criteria for fibromyalgia and measurement of symptom severity. Arthritis Care Res (Hoboken).

[30] Galvez-Sánchez CM, de la Coba P, Duschek S, Reyes Del Paso GA, Reliability. Factor structure and predictive validity of the widespread Pain Index and Symptom Severity Scales of the 2010 American College of Rheumatology Criteria of Fibromyalgia. J Clin Med. 2020;9. 10.3390/jcm9082460 PMC7464133 32752048

[31] Luque-Suarez A, Martinez-Calderon J, Falla D (2019). Role of kinesiophobia on pain, disability and quality of life in people suffering from chronic musculoskeletal pain: a systematic review. Br J Sports Med.

[32] Lin I, Wiles L, Waller R, Goucke R, Nagree Y, Gibberd M (2020). What does best practice care for musculoskeletal pain look like? Eleven consistent recommendations from high-quality clinical practice guidelines: systematic review. Br J Sports Med.

[33] Briggs AM, Slater H, Van Doornum S, Pearson L, Tassone EC, Romero L (2022). Chronic primary or secondary Noninflammatory Musculoskeletal Pain and disrupted sexual function and Relationships: a systematic review. Arthritis Care Res (Hoboken).

[34] Dzakpasu FQS, Carver A, Brakenridge CJ, Cicuttini F, Urquhart DM, Owen N (2021). Musculoskeletal pain and sedentary behaviour in occupational and non-occupational settings: a systematic review with meta-analysis. Int J Behav Nutr Phys Act.

[35] Huguet A, Tougas ME, Hayden J, McGrath PJ, Stinson JN, Chambers CT (2016). Systematic review with meta-analysis of childhood and adolescent risk and prognostic factors for musculoskeletal pain. Pain.

[36] Valentijn PP, Tymchenko L, Jacobson T, Kromann J, Biermann CW, AlMoslemany MA (2022). Digital Health Interventions for Musculoskeletal Pain Conditions: systematic review and Meta-analysis of Randomized controlled trials. J Med Internet Res.

[37] Coles ML, Weissmann R, Uziel Y (2021). Juvenile primary Fibromyalgia Syndrome: epidemiology, etiology, pathogenesis, clinical manifestations and diagnosis. Pediatr Rheumatol.

[38] Lubrano E, Scriffignano S, Morelli R, Perrotta FM (2021). Assessment of widespread and Extraarticular Pain in Psoriatic Arthritis: a case-control study. J Rheumatol.

[39] Dudeney J, Law EF, Meyyappan A, Palermo TM, Rabbitts JA (2019). Evaluating the psychometric properties of the widespread Pain Index and the Symptom Severity scale in youth with painful conditions. Can J pain = Rev Can la douleur.

[40] Elkana O, Falcofsky AK, Shorer R, Bar-On Kalfon T, Ablin JN (2019). Does the cognitive index of the symptom severity scale evaluate cognition? Data from subjective and objective cognitive measures in fibromyalgia. Clin Exp Rheumatol.

[41] De Sousa AP, de Arruda GT, Pontes-Silva A, de Souza MC, Driusso P, Avila MA. Measurement properties of the brazilian online version of the Fibromyalgia Rapid Screening Tool (FiRST). Adv Rheumatol (London, England). 2022;62:39. 10.1186/s42358-022-00271-2 PMC9628418 36316763

[42] Mokkink LB, Prinsen CA, de Patrick DLJALMBHC (2019). COSMIN Study Design checklist for patient-reported outcome measurement instruments.

[43] Pauli J, Starkweather A, Robins JL (2019). Screening tools to predict the development of chronic low back Pain: an integrative review of the literature. Pain Med.

[44] Akin-Akinyosoye K, James RJE, McWilliams DF, Millar B, das Nair R, Ferguson E (2021). The Central Aspects of Pain in the knee (CAP-Knee) questionnaire; a mixed-methods study of a self-report instrument for assessing central mechanisms in people with knee pain. Osteoarthr Cartil.

[45] Noori SA, Rasheed A, Aiyer R, Jung B, Bansal N, Chang K-V (2020). Therapeutic Ultrasound for Pain Management in Chronic Low Back Pain and Chronic Neck Pain: a systematic review. Pain Med.

[46] Botros J, Gornitsky M, Samim F, der Khatchadourian Z, Velly AM (2022). Back and neck pain: a comparison between acute and chronic pain-related Temporomandibular Disorders. Can J pain = Rev Can la douleur.

[47] Wolfe F, Walitt B, Perrot S, Rasker JJ, Häuser W (2018). Fibromyalgia diagnosis and biased assessment: sex, prevalence and bias. PLoS ONE.

