# Supplementary material for: Do the instruments used to assess fibromyalgia symptoms according to American College of Rheumatology criteria generate similar scores in other chronic musculoskeletal pain?
Source: BMC Musculoskelet Disord. 2023 Jun 7;24:467. doi: 10.1186/s12891-023-06572-x (PMC10245398; doi:10.1186/s12891-023-06572-x)
Supplement: Supplementary file 1 — Supplementary Material 1 [file 12891_2023_6572_MOESM1_ESM.docx]

Chronic pain

Booklet developed by Mariana Arias Avila Vera for the Project Chronic low back pain: physiotherapeutic care associated with pain neuroscience education

2018

Material development

• This material was developed based on materials available on the websites www.pesquisaemdor.com.br and www.retrainpain.org/portugues and on the book Butler DS, Moseley L. Explain Pain. Australia. NOI Group Publishing, 2003

• For the preparation of the booklet, the following article was used: Mendez SP, Sá KN, Araújo PCS, Oliveira IAVF, Gosling AP, Baptista AF. Development of an educational booklet for people with chronic pain. Rev. Dor, 18(3):199-211, 2017

• The booklet “Pain does not overcome me, I know how to live” (Godoy AG, Tessarin BM, Gonçalves D, Silva GC, Souza TA, Silva VF, 2017) developed by students of the General Physiotherapy Internship at UFSCar was adapted to the context of extension project.

What is the pain?

Pain is normal, it's a signal, like an alarm that works to protect our body. When we cut our finger, our body detects something different from normal, and this information travels through the nerves and reaches our brain, which interprets and evaluates what the information is trying to say. To do this, he gathers all the other information he has about us (physical, emotional, health, sleep, etc.), and makes a decision: does our alarm need to go off?

NOT EVERY TIME WE HAVE ANY PART HURT, WE WILL HAVE PAIN NOT EVERY TIME WE HAVE PAIN MEANS WE ARE HURT

But how so?

• Athlete Kerri Strug won a gold medal at the 1996 Olympic Games in Atlanta. During the competition, she suffered a fracture, but continued to compete, and she reported no pain during her performance.

• When we cut our finger with paper, we feel a lot of pain, even with a small wound that does not offer any great danger to our body.

• Therefore, test results do not always indicate that there is a very big problem with you, it just describes how your body is inside. The exam often indicates something that is unrelated to your pain, and in the place that hurts, there is nothing “wrong”.

The role of the brain

• Who says if and how much it hurts is always the brain, taking into account all the other things we are feeling.

• If we're scared, anxious, stressed, sleeping badly, waking up tired, without doing physical activity on a regular basis, the pain we'll feel when we get hurt will probably be much greater.

• This cycle could go on for a long time if we don't intervene in it. This cycle does us no good. The repetition of this cycle (“bad” feelings - pain - “bad” feelings) will cause changes in our nerves and brain, and these changes can disrupt our alarm.

Remember our alarm?

• Our alarm should go off only when there is something abnormal... But now it goes off even when everything is normal.

• It's like that car alarm that goes off when a bus passes in the street, or when someone passes close to it.

• And it bothers the neighbors (ie the other body parts). Therefore, those who have chronic pain are more sensitive to pain in other places that they did not feel pain in the beginning.

Good news

• We can be like the mechanic who sets the car alarm: we can help the body set our alarms so they work properly again!

• As? Exercising, meditating, relaxing, hanging out with friends, feeling less afraid of our pain, doing more activities!

• Living with pain is not easy, several doubts and concerns arise. Here are some tips on how we can go through this experience more lightly.

Tips

• Acceptance: When we have pain, we enter a cycle: pain generates fear and worry, which limits us in our work and activities of our daily lives. When we accept that pain exists, it becomes easier to seek treatment. Accepting is having greater control and knowing how to improve the situation.

• Acceptance is a difficult and long process. Don't cover yourself, be patient with yourself. Not every day will be good, but even on bad days there are good things. Remember: accepting is also recognizing your limitations and being at peace with them!

Tips

• You are not alone: ​​around 1 in 5 people worldwide have pain; 1 in 10 are diagnosed with chronic pain each year.

Tips

• Sleep: Sleeping poorly makes you more tired, moody, and this makes our alarms go haywire. Remember? Lack of rest causes our body to produce substances that increase our pain, makes us more irritable and can make us more depressed or anxious.

• To find out if you're sleeping well, answer the following questions:

Do you have difficulty falling asleep at night?

Do you wake up at night and stay awake?

Do you wake up the next day feeling like you haven't rested?

• If you answered YES to any of these questions, you're going to need to think of ways to improve your sleep!

Tips

• How to improve sleep?

• Make your bedroom a relaxing place for you

• Make an effort to sleep at similar times: choose a time to go to bed daily, and set times to wake up, including on weekends

• Try not to use a computer, cell phone or tablet close to bedtime: the light that electronics emit can disturb your brain to understand that it's time to sleep!

• Try to make the room dimmer.

• Do some physical activity during the day (eg walking)

• Avoid naps during the day

• Do not eat heavy meals close to bedtime

• If you smoke, try to reduce the number of cigarettes during the day, trying to avoid smoking at night.

What can influence the pain?

• Tiredness • Family problems

• Financial problems • Problems at work

• Insomnia • Worries

• Depression • Weight gain

• Stress • Sadness

• Irritation • Anger

Tips

• Emotional: The emotional part is very important when we think about pain. So, when you are going through a difficult situation that provokes bad emotions in you (anger, worry), try to control your reaction:

• Realize your breathing: Try not to get out of breath, try to keep your breath calm;

• Activities: either in a group, or a meditation or yoga activity, can help

• Exercise: try to practice regularly;

• Sleep: try to improve the quality of your sleep;

• Pleasant activities: courses, outings, trips, programs that are good for you should always be done;

• Think positive: try to see the bright side of the situation. This doesn't always sound easy, but it's very important that you try. This practice will help you, over time, to deal with these situations more easily.

Tips

• Physical Exercise and Pain: We tend to stay still when we are in pain. Rest is necessary, but it should not be continuous!

Standing still can leave us in more pain!

Tips

• To start being more active, you need to be patient. Go slowly, respecting your limits, understanding when your pain appears.

• Simple activities help expend energy and reduce pain. The discomfort they can generate is NOT pain, and not even a reason to give up!

• To be able to do this, you may need to change your habits!

• Change one habit at a time.

• Think of solutions that you can do (walk 15 minutes a day) and not outlandish (spend 4 hours a day at the gym).

• Do not give up! It may not work the first few times; think of different strategies, and try as many times as necessary!

Tips

• How to start the exercises?

• Remember, exercise can bring many benefits to those in pain! The body, after exercise, releases substances that help control pain (more or less “relatives” of morphine) and leave the body a little anesthetized.

• Getting started is difficult, and must be done slowly. Exercise intensity should only be adjusted if you feel comfortable. Respect your limits!

• Do exercises that you enjoy, in pleasant places.

• Try to make exercise part of your routine, with scheduled days and times.

• Vary your exercises and have a plan B (if it rains, you can do your exercises at home)

• After exercising, slight muscle soreness may appear! Do not panic! It means your body worked!

Tips

• Pain and Relationships: When we are in pain, we are more irritable and complain more often. The people who live with us are affected by this change in our behavior. This has consequences both professionally and personally.

• It is important that you seek help, and try to express yourself adequately. Look for someone you trust, who will understand what's going on. Try to be kind when asking for help.

• If you live with someone who has chronic pain, try to hear what that person has to say. Try to welcome this person as you would like to be welcomed.

Tips

• Take a deep breath: Breathing helps us to relax, and this can help with pain control.

• Relaxing in moments of great irritation is something very difficult, but a deep and careful breathing helps a lot!

• Don't let pain get in the way of your relationships!

Recovery

• Don't let pain interfere with your life too much!

• Seek better quality sleep;

• Try to think more positive thoughts - these can help with pain management

• Practice relaxation;

• Be patient and set short-term goals

• Insert breaks in your daily life activities to reduce overload

• Exercise and be more active

• Build strong foundations from friends, family and healthcare professionals

• Do not give up!
